# Quantitative evaluation of the performance of different deformable image registration algorithms in helical, axial, and cone‐beam CT images using a mobile phantom

**DOI:** 10.1002/acm2.12246

**Published:** 2018-02-15

**Authors:** Imad Ali, Nesreen Alsbou, Justin Jaskowiak, Salahuddin Ahmad

**Affiliations:** ^1^ Department of Radiation Oncology University of Oklahoma Health Sciences Center Oklahoma City OK USA; ^2^ Department of Engineering and Physics University of Central Oklahoma Edmond OK USA

**Keywords:** axial CT, cone‐beam CT, CT‐number distribution, deformable image registration, helical CT, motion artifacts

## Abstract

The goal of this project is to investigate quantitatively the performance of different deformable image registration algorithms (DIR) with helical (HCT), axial (ACT), and cone‐beam CT (CBCT). The variations in the CT‐number values and lengths of well‐known targets moving with controlled motion were evaluated. Four DIR algorithms: Demons, Fast‐Demons, Horn‐Schunck and Lucas‐Kanade were used to register intramodality CT images of a mobile phantom scanned with different imaging techniques. The phantom had three water‐equivalent targets inserted in a low‐density foam with different lengths (10–40 mm) and moved with adjustable motion amplitudes (0–20 mm) and frequencies (0–0.5 Hz). The variations in the CT‐number level, volumes and shapes of these targets were measured from the spread‐out of the CT‐number distributions. In CBCT, most of the DIR algorithms were able to produce the actual lengths of the mobile targets; however, the CT‐number values obtained from the DIR algorithms deviated from the actual CT‐number of the targets. In HCT, the DIR algorithms were successful in deforming the images of the mobile targets to the images of the stationary targets producing the CT‐number values and lengths of the targets for motion amplitudes <20 mm. Similarly in ACT, all DIR algorithms produced the actual CT‐number values and lengths of the stationary targets for low‐motion amplitudes <15 mm. The optical flow‐based DIR algorithms such as the Horn‐Schunck and Lucas‐Kanade performed better than the Demons and Fast‐Demons that are based on attraction forces particularly at large motion amplitudes. In conclusion, most of the DIR algorithms did not reproduce well the CT‐number values and lengths of the targets in images that have artifacts induced by large motion amplitudes. The deviations in the CT‐number values and variations in the volume of the mobile targets in the deformed CT images produced by the different DIR algorithms need to be considered carefully in the treatment planning for accurate dose calculation dose coverage of the tumor, and sparing of critical structures.

## 1 INTRODUCTION

Deformable image registration (DIR) provides useful tools in radiation therapy where images from different modalities can be deformed and registered to account for anatomical variations because of patient motion, organ filling, tumor growth with time or shrinkage because of response to treatment with radiation therapy.^1^ DIR has potential applications in image registration, segmentation and dose mapping that can enable the performance of adaptive radiation therapy (ART) by considering anatomical variations in order to obtain conformal dose coverage of tumors and sparing of organs‐at‐risk.^2^, ^3^, ^4^, ^5^, ^6^ ART provides the tools to use updated computed tomographic (CT) images such as kV‐cone‐beam CT (CBCT) obtained from daily patient setup and tumor localization with image‐guided radiation therapy (IGRT) to perform more frequent updates on the planning‐target‐volume (PTV) and other critical structures considering anatomical variations due to tumor response to treatment.^7^ These variations in patient anatomy can be accounted for in reasonable time by considering auto‐segmentation techniques that can use voxel‐by‐voxel deformation of the patient CT images used in treatment planning to current CBCT images obtained from daily patient setup.^8^ Patient motion leads to variations in patient anatomy inter‐ and intrafractions where ART will provide tools to manage the geometric and dosimetric discrepancies between patient CT simulation and treatment planning and dose delivery.^2^ The dose variations due to changes in patient anatomy can be evaluated from the deformed initial dose maps on a voxel‐by‐voxel basis using DIR algorithms. Furthermore, new treatment plans using current CT images acquired daily can be generated and delivered as the treatment course progresses considering variations in patient anatomy to achieve ART.

Patient motion may induce significant artifacts in CT images obtained from the simulation CT, which can affect accuracy of the shape and volume of tumor targets outlined for treatment planning.^9^, ^10^ For example, motion causes the blurring of the edges of mobile targets affecting the accuracy for determination of the boundary of the targets in the CT images with strong motion artifacts.^11^, ^12^, ^13^ In addition, motion artifacts cause variations in CT‐number values invalidating the accuracy of the values of electron density of the mobile target and thus the accuracy of dose calculation.^14^ Different techniques are used to manage patient motion both during simulation CT imaging and dose delivery.^15^ In simulation CT imaging, patient motion is managed by breath holding technique during scanning or by acquiring projection images at different motion phases when the patient is scanned in synchrony with a breathing signal and the projections are sorted in different motion phases to obtain 4D‐CT images.^16^ At the stage of dose delivery, patient motion management includes breath hold technique during irradiation or beam gating when the beam is held on if the motion signal is synchronized with the selected breathing phase window; and is held off outside the gating window.^17^

The goals of this project are to investigate quantitatively the performance of different DIR algorithms with helical CT (HCT), axial CT (ACT), and CBCT images by evaluating the variations in the CT‐number values and lengths of mobile targets inserted in a thorax phantom moving with controlled motion patterns that simulates tumor motion in lung. Four DIR algorithms: Demons,^18^, ^19^ Fast‐Demons,^20^, ^21^ Horn‐Schunk,^22^ and Lucas‐Kanade^23^, ^24^ from the DIRART software were selected to register CT images of a phantom which moved with controlled motion patterns. The variations in the volumes and CT‐number values of the mobile targets obtained from deformed images were quantified.

## 2 MATERIALS AND METHODS

### 2.A Phantom setup and imaging

**Figure 1:**
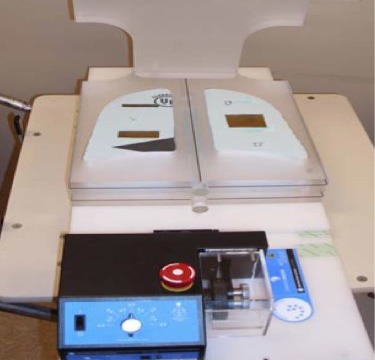
Mobile phantom system.

Three tissue‐equivalent targets were inserted in a thorax phantom that was mounted on a mobile platform (Standard Imaging, Inc., Middleton, WI, USA). The three targets small, medium, and large had well‐known shapes and volumes with lengths of 10, 20, and 40 mm in the direction of motion were embedded in low‐density foam simulating lung‐tissue as shown in Fig. 1. The phantom moved along the *Y*‐axis in the superior‐inferior direction with adjustable motion amplitudes and frequencies. In this experiment, the phantom was imaged with different techniques, helical, axial, and cone‐beam CT while it was stationary and moving. The phantom moved during imaging with different motion amplitudes in the range (0–20 mm) and frequencies (0.1–0.5 Hz). Helical CT imaging was performed with a simulation CT (GE Discovery‐CT‐590RT, General Electric Healthcare, Milwaukee, WI, USA) using a thorax technique with a pitch of 1.375; effective field‐of‐view of 65 cm diameter, axial reconstruction matrix of 512 × 512 pixels, slice thickness of 2.5 mm; 120 kVp; 440 mA and 1 s rotation time. Similar parameters were used for axial CT imaging. The cone‐beam CT images were obtained from an on‐board imaging (OBI) system mounted on a TrueBeam STX machine (Varian Medical Systems, Inc., Palo Alto, CA, USA). The OBI was made from a kV x‐ray source and a flat‐panel detector that had an effective imaging area of nearly 30 × 40 cm^2^ (PaxScan^®^ 4030CB) with a spatial resolution of 1024 × 768 pixels. Two CBCT‐imaging modes including full‐fan and half‐fan were used. The full‐fan CBCT images covered a region of 25 cm diameter and 15 cm thickness where the radiographic projections were obtained by rotating the gantry about 180^o^ around the phantom, while the half‐fan CBCT images were acquired by a full circle rotation around the phantom in order to cover a wider region of 50 cm diameter and 17 cm thickness. The imaging parameters for the half‐fan CBCT were 2 mm slice thickness, 125 kVp and 264 mAs, while 2 mm slice thickness, 100 kVp and 146 mAs were used for the full‐fan CBCT.

### 2.B Deformable image registration algorithms

The DIRART research software developed at the University of Washington‐Saint Louis was used in this study.^1^ It includes a variety of DIR algorithms coded in the MATLAB software (MathWorks, Inc., Natick, MA, USA) and has software tools to perform important functions in implementing adaptive radiation therapy.^3^ Four DIR algorithms: Demons,^18^, ^19^ Fast‐Demons,^20^, ^21^ Horn‐Schunck,^22^ and Lucas‐Kanade^23^, ^24^ were used to register the CT images of a mobile phantom along with CT images of the stationary phantom. These algorithms represent a variety of autonomous intensity‐based algorithms DIR to solve the optical flow equation. The Demons algorithm extends the diffusion model into an optical flow equation. Optical flow involves the displacements between two image sets during a temporal sequence that is represented in intensity variations.^18^, ^19^ The Demons algorithm follows similar theoretical physics principles as the Maxwell's demons in fluids. The diffusion model in image registration is based on the physics of thermodynamics of gases or fluids that is applied in the information theory for mutual entropy minimization techniques. The Demons algorithm uses two images that are considered to be bounded by semipermeable membranes. Then, one image diffuses through the other as a deformable grid that is driven by ‘demons’ forces on the perimeter of the image. In a diffusing model, the demons force at each point is sampled at the image boundary. The forces collectively diffuse the moving image through the boundary of the static image and remain constantly decreasing in magnitude until the images are aligned in the same coordinate space. The Demons algorithm needs an additional constraint to solve the aperture problem.^22^ The optical flow is calculated in two steps: (a) the instantaneous optical flow is computed for every point within the static image, and (b) the deformation field is regularized by smoothing with a Gaussian filter.^18^ The Fast‐Demons algorithm explicitly takes into account Newton's third law of motion in combination with the diffusing model used by the Demons algorithm.^20^, ^21^ In Fast‐Demons, the demons forces allow additionally the moving object to diffuse through the static or reference image.^21^ This active force in the Fast‐Demons algorithm serves as an amplifying factor in the overall force applied in the Demons algorithm.^20^, ^21^

The Horn‐Schunck algorithm represents optical flow as variations in the image intensity by an apparent distribution of velocities from the movement of different image voxels.^22^ A global smoothness constraint is imposed to satisfy the aperture problem where the number of independent variables is larger than the number of independent linear equations.^22^ The aperture problem is solved by considering an optical flow constraint equation which is convolved with a Gaussian low‐pass filter where two main approaches are used. The global methods such as the Horn‐Schunck algorithm employ regularizing smoothness terms, whereas the local differential methods such as Lucas‐Kanade assume spatial constancy to solve the problem of non‐uniqueness. The Lucas‐Kanade algorithm uses three key assumptions for the optical flow equation that include: (a) intensity is constant between the two image sets, (b) the voxels move small displacements and (c) neighboring voxels move with same velocity.^23^, ^24^ This algorithm considers small motions of the different voxels in the images, thus the motion of more than the range of a pixel will invalidate image registration. The image intensity must also remain constant and a pixel should move like its neighbors for the Lucas‐Kanade method to work successfully. For large motion of the voxels, the resolution of the images is reduced. Local optical flow methods such as the Lucas‐Kanade algorithm are more robust with noisy images; however, the velocity or DVFs are less dense than the global methods such as the Horn‐Schunck technique. A multigrid approach of four stages was used to sample down the images by a factor of 2 at each stage in sequence from low to high resolution. Multiple passes (2,3,4,5 passes) were used where in each pass the registration was performed with smaller number of iterations (10,20,30,40 iterations) with a multiple grid Gaussian filter and smoothing parameter of 3 during iterations and (0.5 0 0) after passing. The DIRART software provides the tools to perform validation test for the different DIR algorithms that include inverse consistency and Jacobian analysis which were within acceptable limits in this study.

## 3 RESULTS

### 3.A CBCT images

**Figure 2:**
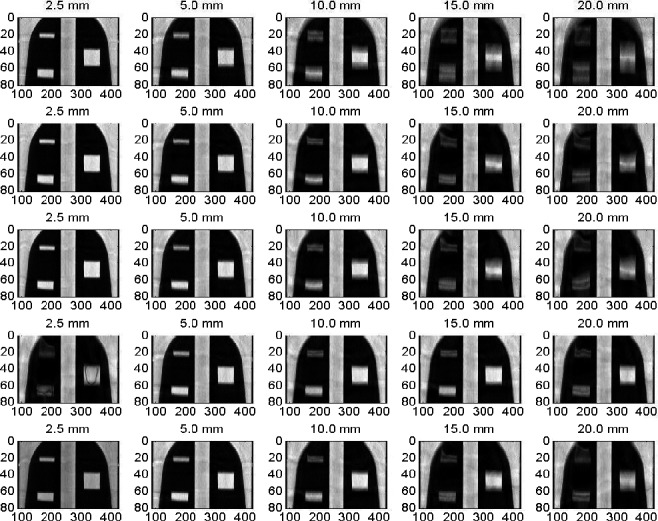
Coronal views from the CBCT images reconstructed at the central level of the targets embedded in the thorax phantom with various motion amplitudes and DIR algorithms where each column represents one motion amplitude as indicated. The images in the first row represent the CBCT of the moving targets and the second to fifth rows represent the deformed imaged using the Demons, Fast‐Demons, Horn‐Schunck, and Lucas‐Kanade, respectively.

Figure 2 shows coronal views of the mobile targets (small, medium, large) registered to the CBCT images of the stationary phantom used as a reference image to which the moving images have been registered using different deformable image registration algorithms. The deformed CBCT images of the mobile phantom are shown for several motion amplitudes (2.5–20 mm) and four deformable image registration algorithms: (a) Demons, (b) Fast Demons, (c) Horn‐Schunck, and (d) Lucas‐Kanade. Increasing motion amplitudes enhanced image artifacts in the CBCT images where the lengths of the mobile targets were elongated along the direction of motion. The motion artifacts at lower amplitudes were suppressed and image quality was improved in the deformed CBCT images. All DIR algorithms were able to reproduce the shapes and volumes of the three targets in the deformed CBCT images of the mobile phantom at small and intermediate motion amplitudes. However, at large motion amplitudes, the shapes and volumes of the small mobile target were not reproducible at the largest motion amplitude of 20 mm used in this study. The Demons and Fast‐Demons algorithms behaved well at small motion amplitudes and failed at large motion amplitudes. The Horn‐Schunck algorithm performed well over a large range of motion amplitudes. The Horn‐Schunck technique considers spatial as well as smoothing components. It employs a global method that computes the optical flow velocity based on a neighborhood average and then filters the results with a Gaussian filter. Therefore, Horn‐Schunck penalizes large errors in the intensity constancy. The Lucas‐Kanade performance was in between the previous DIR algorithms where it employs local spatial components only.

**Figure 3:**
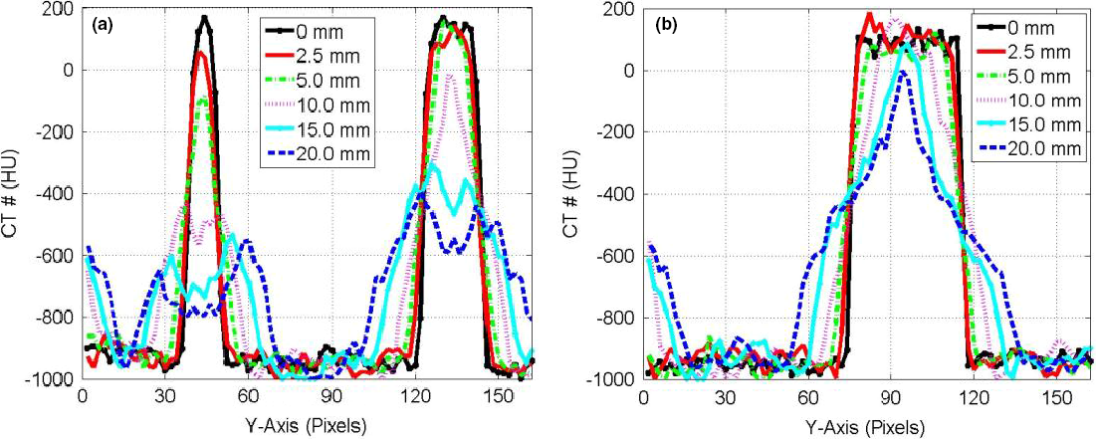
CT‐number profiles for the small and medium targets (a) and larger target (b) in CBCT images along the direction of motion in pixels (2.0 mm) for motion amplitudes in the range 0–20 mm.

**Figure 4:**
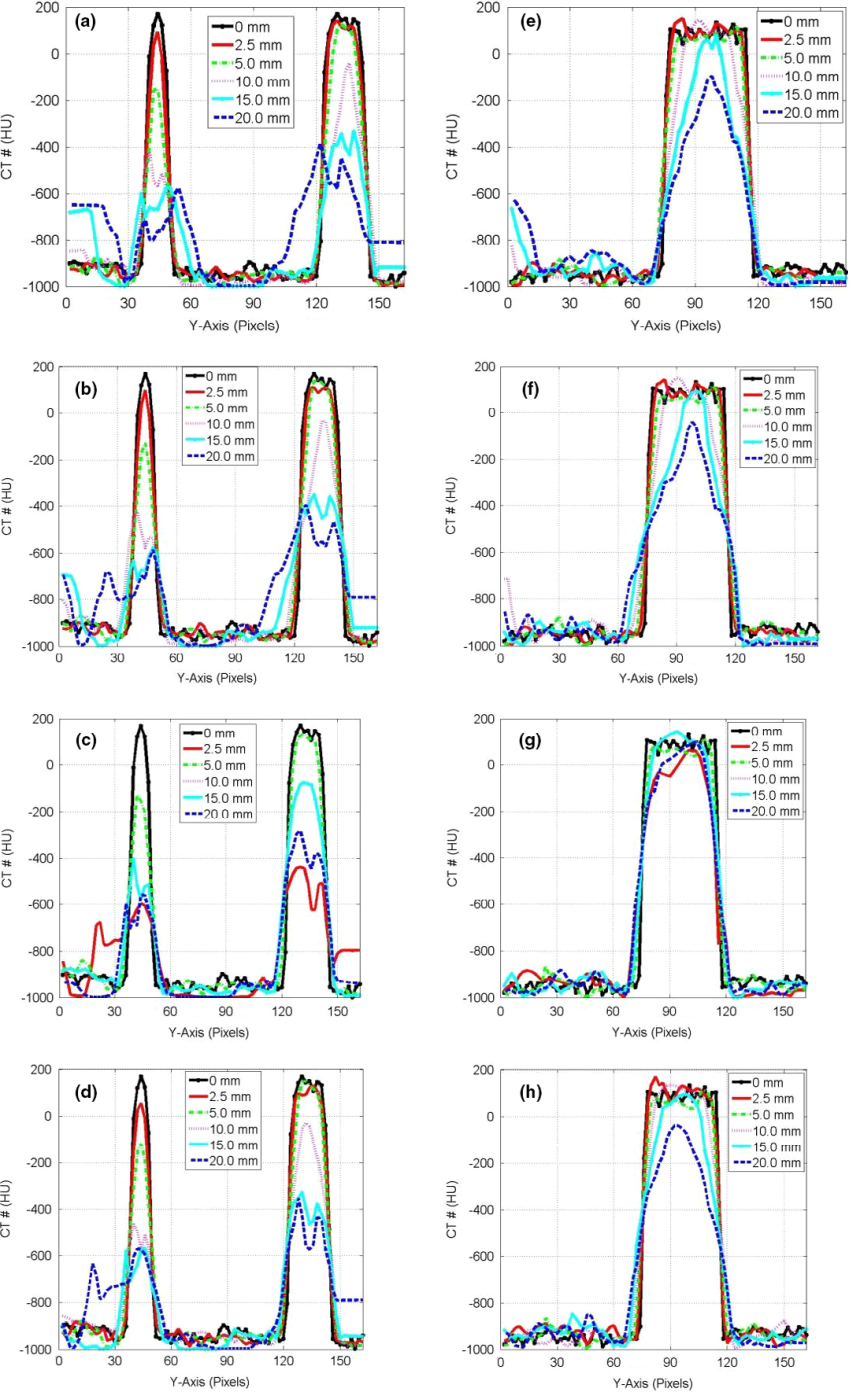
CT‐number profiles along the direction of motion in pixels (2.0 mm) for the deformed images by the different algorithms (a) Demons, (b) Fast‐Demons, (c) Horn‐Schunck, and (d) Lucas‐Kanade in the first column for the small and medium targets in CBCT images. (e,f,g,h) in the second column are the CT‐number profiles for the large target as in the first column.

Figure 3 shows the CT‐number profiles from the stationary and mobile CBCT images of the three targets: small, medium, and large along the direction of motion. Two major effects were pronounced in the CT‐number profiles of the mobile targets: (a) the CBCT‐number distributions spread‐out more beyond the actual lengths of the stationary targets and (b) the level of the CT‐number values decreased as the motion amplitude increased for the CBCT profiles of the targets. Figure 4 shows the CT‐number profiles for CBCT images of the small and medium stationary and mobile targets along the superior‐inferior direction parallel to the phantom motion (*Y*‐axis). Four DIR algorithms were used that included the Demons in (a‐b), Fast‐Demons in (c‐d), Horn‐Schunck in (e‐f), and Lucas‐Kanade in (g‐h) to correct motion artifacts with the phantom moving with the indicated motion amplitudes. The different DIR algorithms produced the physical length and shape of the original target (stationary target) for small and medium at motion amplitudes ≤15 mm. As the motion amplitude increased, the DIR algorithms failed to reproduce the length of the original target for motion amplitudes 17.5 and 20.0 mm, respectively. While the actual shapes and volumes of the different targets were reproduced by most of the DIR algorithms, there were large discrepancies between the CT‐number values obtained from the different DIR algorithms and the corresponding CT‐numbers of the stationary targets. For example, the differences in the CT‐numbers increased gradually from 400 HU at small motion amplitude up to 1000 HU for large motion amplitudes for the small target. For the larger target, the difference in the CBCT profile between the deformed and stationary images ranged from nearly 200 to 700 HU over the range of motion amplitudes from 5 to 20 mm. This indicates that the CT‐number values produced by the different DIR algorithms might not be valid for dose calculation algorithms used to correct tissue heterogeneities in treatment planning particularly at large motion amplitudes in CBCT images.

### 3.B HCT images

**Figure 5:**
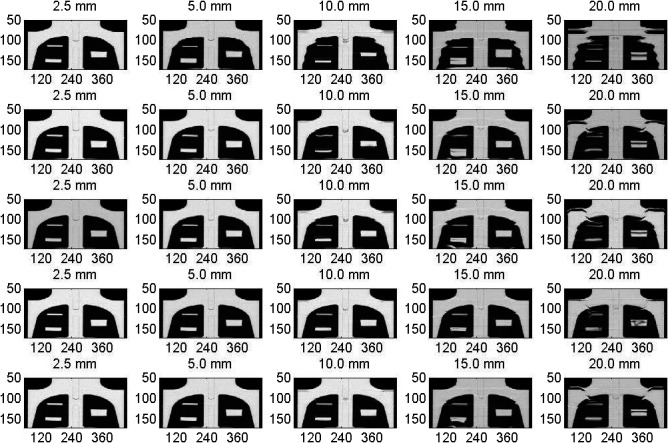
Coronal views from HCT images reconstructed at the central level of the targets embedded in the thorax phantom with various motion amplitudes and DIR algorithms with each column representing one motion amplitude as indicated. The images in the first row represent the HCT of the moving targets and second to fifth rows represent the deformed imaged using the Demons, Fast‐Demons, Horn‐Schunck, and Lucas‐Kanade, respectively.

Figure 5 shows coronal views from HCT images for the small, medium, and large targets obtained from the different DIR algorithms. Similar to CBCT, four DIR algorithms were used to deform the mobile HCT images and to reproduce images that matched with the stationary images of the targets. The coronal views in the first row (a,b) represented the images obtained from Demons, the second row (c,d) from Fast‐Demons, the third row (e,f) from Horn‐Schunck, and the fourth row (g,h) from the Lucas‐Kanade with different motion amplitudes as indicated. The shapes and volumes of the targets were reproducible even at large motion amplitudes (20 mm) particularly for the large mobile target (40 mm). The small and medium targets showed irregular distortion in the shapes such as shrinkage or elongation depending on their motion phases during HCT in contrast with CBCT images which showed a regular increase in the length of the mobile targets with motion amplitudes along the direction of motion. This was due to the interplay between the motion of imaging table and the mobile phantom in HCT imaging. If the mobile targets moved along the direction of the couch motion, they became elongated and if they moved opposite to the couch motion they shrank along the direction of motion. Most of the DIR algorithms were able to deform the distorted mobile targets and to reproduce the actual shapes in HCT imaging. However, the Fast‐Demons algorithm had the least performance compared to the other algorithms investigated in this study.

**Figure 6:**
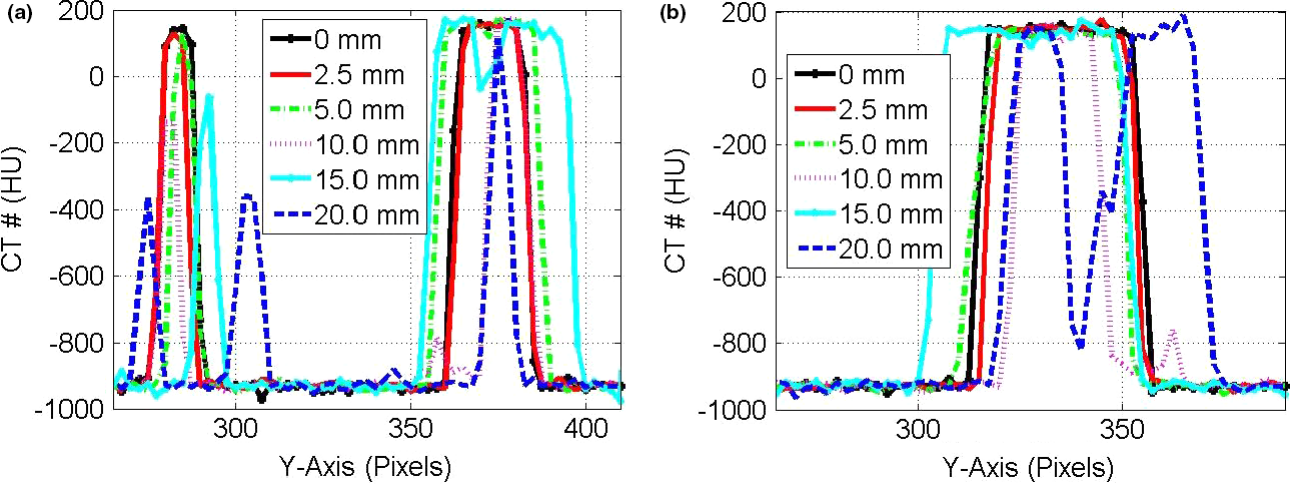
CT‐number profiles for the small and medium targets in HCT images using different DIR algorithms: (a) and large target (b) along the direction of motion in pixels (2.5 mm) with different motion amplitudes (0–20 mm) from HCT images.

Figure 6 shows the CT‐number profiles of the stationary and mobile targets along the direction of motion (*Y*‐axis) for the small and medium (a) and large (b) targets in HCT images. The CT‐number distributions in HCT were affected by motion with image artifacts that included: (a) displacement in the position of the mobile target, (b) elongation or shrinkage in the length of the mobile targets depending on the phase of motion during imaging, and (c) variation in the CT‐number level. The CT‐number distributions of the small and medium targets were distorted more than that of the large target considering the same motion amplitude.

**Figure 7:**
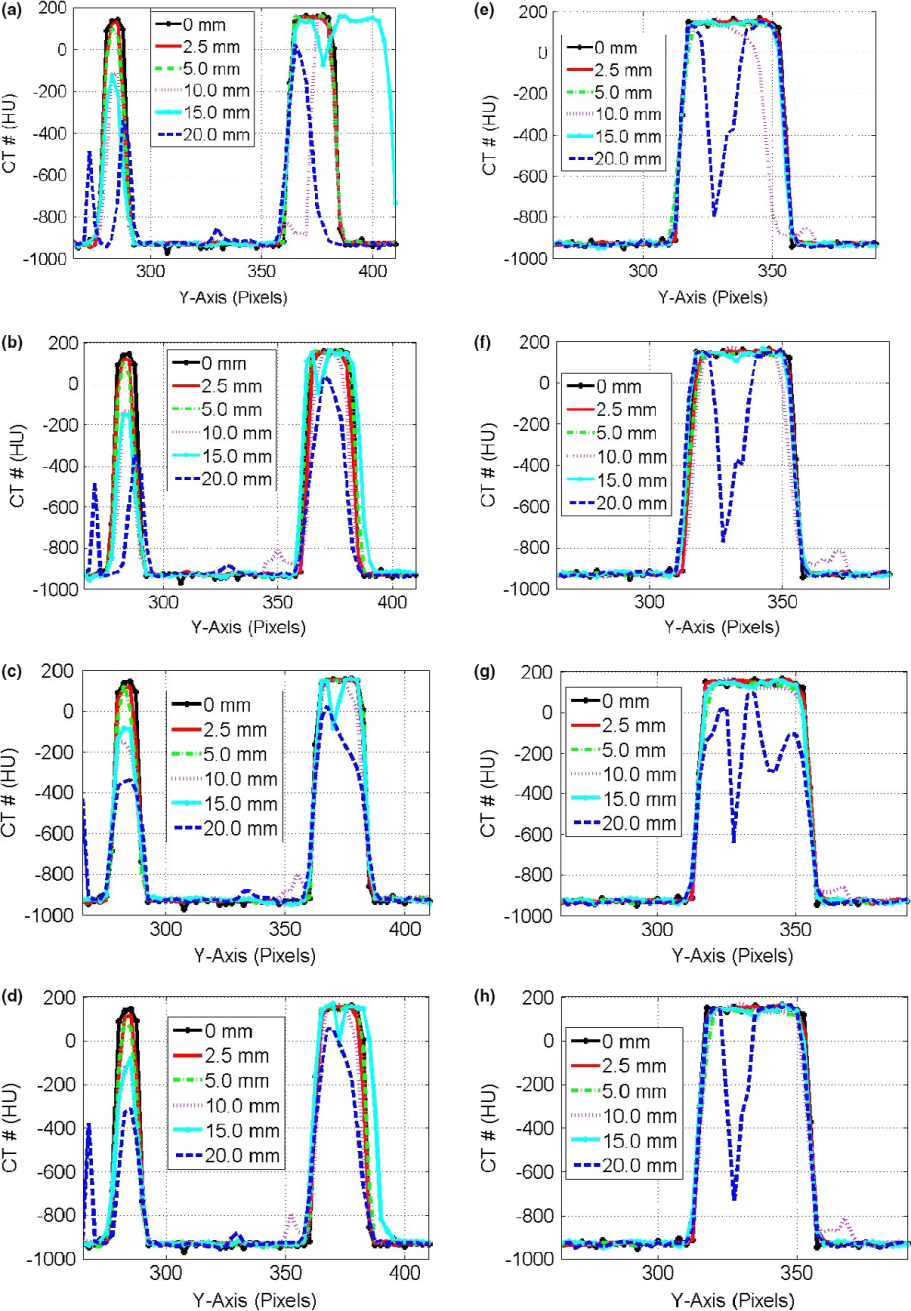
CT‐number profiles along the direction of motion in pixels (2.5 mm) for the deformed images by the different algorithms (a) Demons, (b) Fast‐Demons, (c) Horn‐Schunck, and (d) Lucas‐Kanade in the first column for the small and medium targets in HCT images. (e,f,g,h) in the second column are the CT‐number profiles for the large target as in the first column.

Figure 7 shows a comparison of the CT‐number profiles from the stationary targets and the deformed HCT images using the different DIR algorithms with motion artifact along the direction of motion. In HCT, both the CT‐number level and length of the stationary targets obtained from the deformed CT‐number profiles were reproducible up to relatively large motion amplitude: ≤10 mm for the small and medium targets and ≤15 mm for the large target. At large motion amplitudes (17.5 and 20 mm), the image artifacts induced by motion distorted the HCT images significantly and most of the DIR algorithms were not able to reproduce the volume and CT‐numbers of the mobile targets in the deformed images. The different DIR algorithms were able to reproduce both the shape and CT‐number distributions in HCT and their performance was superior compared to CBCT. The shapes and volumes from the deformed HCT images were valid and they can be used to contour the targets in order to define the PTV in the treatment planning process. The CT‐numbers were valid for dose calculations and heterogeneity corrections at low and intermediate motion amplitudes as demonstrated in this study.

### 3.C ACT images

**Figure 8:**
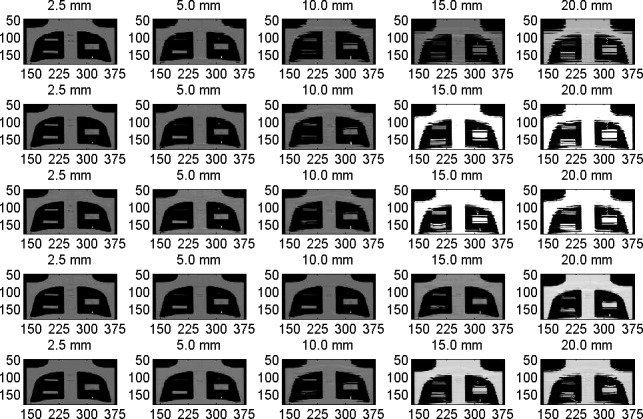
Coronal views from ACT reconstructed at the central level of the targets embedded in the thorax phantom with various motion amplitudes and DIR algorithms where each column represents one motion amplitude as indicated. The images in the first row represent the ACT of the moving targets and second to fifth rows represent the deformed imaged using the Demons, Fast‐Demons, Horn‐Schunck, and Lucas‐Kanade, respectively.

Figure 8 shows coronal views from ACT images produced by the different DIR algorithms of the mobile phantom with the small, medium, and large targets similar to Figs. 2 and 5. Each column represents the variations in the CT‐number distributions with different motion amplitudes. Four DIR algorithms were used where the coronal views in the second row represent the images produced by Demons, the third row by Fast‐Demons, the fourth row by Horn‐Schunck, and the fifth row by the Lucas‐Kanade. In ACT, the image artifacts induced by motion were more predominant compared to the corresponding artifacts in HCT for similar motion parameters of amplitudes and frequencies. This has been attributed mostly to shorter scan times by HCT compared to ACT. For small motion amplitudes of <5 mm, the different DIRs were able to reproduce the shapes and volumes of the different mobile targets because of the small image artifacts induced in the ACT images. As the motion amplitude increased, strong image artifacts were induced by motion in the ACT images which led to splitting in the images of the mobile targets. The different DIRs failed to reproduce the shapes and volumes of the mobile targets in the deformed images particularly for the small target with high CT‐number gradients; and the CT‐number spread‐out distributions to a spatial range, comparable to the length of the target along the direction of motion. The reproducibility of the shapes and volumes of the large target was better than the small and medium targets with large motion amplitudes because image artifacts induced by motion did not distort the shapes of the large target significantly. The Horn‐Schunk and Lucas‐Kanade DIRs generally performed better than the Demons and the Fast‐Demons particularly at large motion amplitudes with strong image artifacts induced by motion.

**Figure 9:**
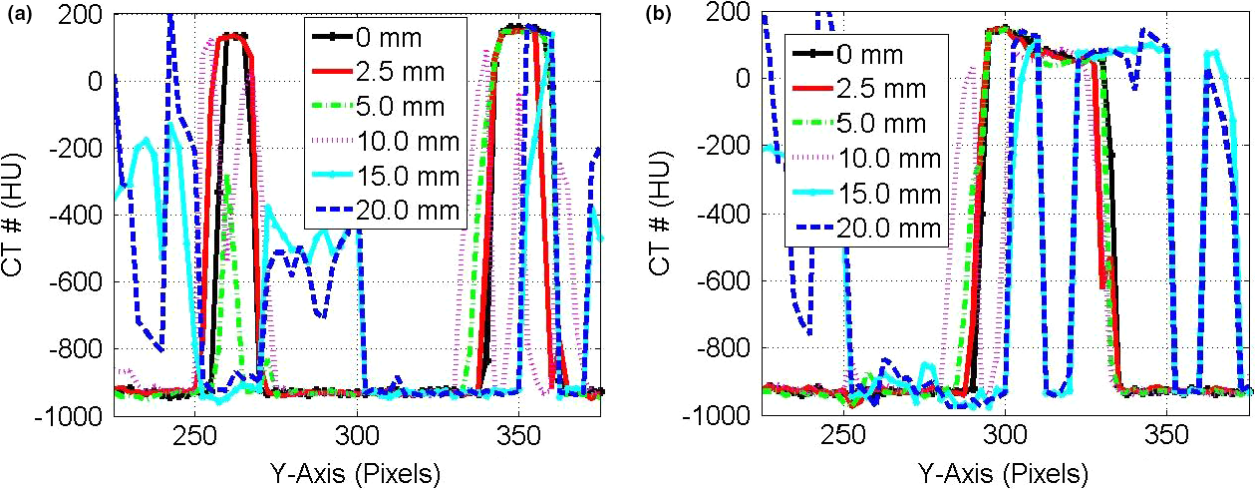
CT‐number profiles for (a) the small and medium targets, and (b) the larger target along the direction of motion for motion in pixel (2.5 mm) amplitudes in the range 0–20 mm from ACT images.

**Figure 10:**
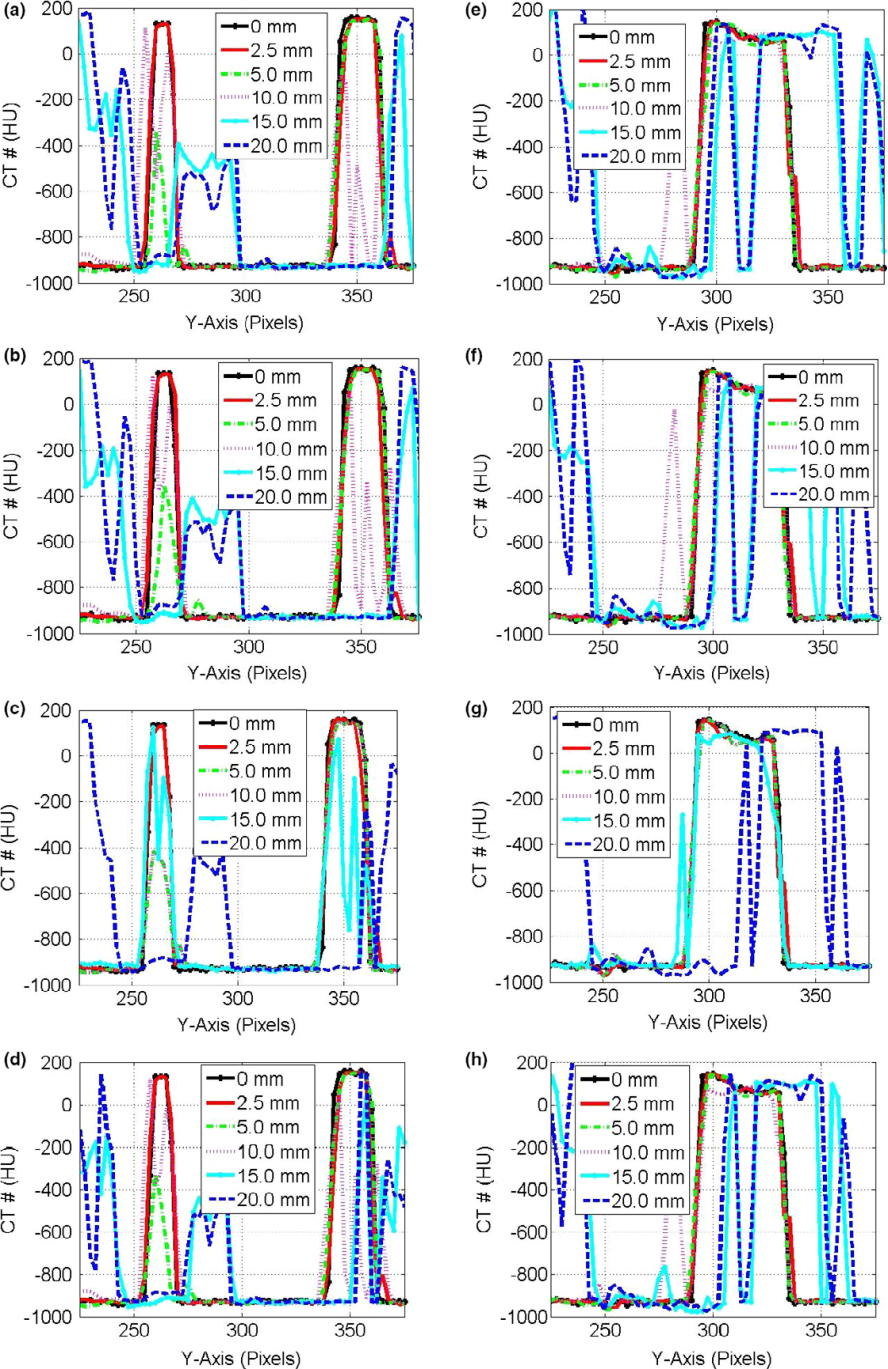
CT‐number profiles along the direction of motion in pixels (2.5 mm) for the small and medium targets in the first column of the deformed HCT images by the different algorithms: (a) Demons, (b) Fast‐Demons, (c) Horn‐Schunck, and (d) Lucas‐Kanade with different motion amplitudes as indicated. The CT‐number profile in (e,f,g,h) in the second column for the large target as in the first column.

Figure 9 shows the profiles of the CT‐number distributions through the center of the target along the direction of motion of the phantom. The profiles represent the variations in the CT‐number distributions with motion amplitudes for the mobile phantom and deformable images produced by the different DIR algorithms. The CT‐number levels and lengths of the mobile targets were reproducible by the deformed CT‐number profiles for low‐motion amplitudes ≤5 mm for the small target, ≤10 mm for the medium target, and <15 mm for the large target. The shape of the targets was distorted at large motion amplitudes because of the strong image artifacts induced by the phantom motion. The different DIR algorithms showed better performances in deforming images of the mobile target to produce the actual CT‐number levels and shapes of the stationary targets in HCT compared to ACT as shown in Fig. 10. This is due to the strong image artifacts in ACT induced by motion artifacts which led to splitting of the different targets at large motions amplitudes.

## 4 DISCUSSION

In this study, an experimental phantom benchmark was designed to investigate quantitatively the performance of different DIR algorithms. This phantom benchmark with well‐known targets has water‐equivalent densities, and specific shapes and volume inserted in low‐density foam where the boundaries of the targets that move with controlled motion are well defined which make quantitative evaluation of the performance of DIR algorithms possible. In contrast, evaluation of the performance of the DIR algorithms in cancer patients is difficult because the actual tumors usually move irregularly, they have different shapes and their densities are similar to surrounding tissues. This study demonstrated that in CBCT, most of the DIR algorithms of the mobile targets were able to produce the shapes and volumes of the mobile targets. However, they did not produce the CT‐number values of the stationary targets in the deformed images. The image artifacts induced by motion were more regular in CBCT imaging where the mobile target elongation increased linearly with motion amplitude. The CT‐number level decreased as motion amplitude increased, while the dependence on motion frequency and phase were negligible. In HCT and ACT, the motion artifacts were irregular where some mobile targets were elongated or shrunk depending on the motion phases during imaging. The DIR algorithms were successful in deforming the images of the mobile targets to the images of the stationary targets producing the CT‐number values, lengths, and shapes of the targets for relatively large motion amplitudes <20 mm in HCT. In ACT, all DIR algorithms produced the actual CT‐numbers and lengths of the stationary targets up to intermediate motion amplitudes <15 mm. The DIR algorithms failed to produce valid shapes, volumes, and CT‐numbers of the mobile targets in the deformed images at large motion amplitudes. Besides the strong dependence on amplitude, motion frequency and phase played important role in the significance of the image artifacts induced by motions. This was represented in the elongations and shrinkage effects of the targets along the direction of motions in HCT and ACT images. The Horn‐Schunck and Lucas‐Kanade which are optical flow‐based algorithms performed better than the Demons and the Fast‐Demons particularly at large motion amplitudes. However, the Demons and the Fast‐Demons that are based on attraction forces converged faster than the optical flow algorithms.

In order to be able to use DIR algorithms in ART, it is crucial to produce the actual shapes, volumes, and the CT‐number values of the mobile targets. This is important to determine accurate tumor volume that is used in treatment planning to define the PTV. However, this study demonstrated that the performance of the different DIR algorithms depends on the motion artifacts and the modality of imaging. For small motion amplitudes, most of the DIR algorithms used in this study were able to reproduce the lengths of the mobile targets along the direction of motion. However, at large motion amplitudes, nearly all four DIR algorithms were not able to produce the shapes and volumes of the mobile targets. Besides the volumes and shapes of the different tumor targets, motion artifacts affected the CT‐number values which were not reproduced in the deformed images by the different DIR algorithms at large motion amplitudes in all CT‐imaging modalities. At small motion amplitudes, the different DIR algorithms performed better in HCT compared to ACT and CBCT. Therefore, the use of the target volumes and CT‐number values obtained from DIR algorithms has to be evaluated carefully before use in treatment planning and dose calculation to correct heterogeneity in radiation therapy.

## 5 CONCLUSIONS

This study used a mobile thorax phantom that has three water‐equivalent targets with well‐known shapes and sizes that are inserted in low‐density material in order to evaluate quantitatively the performance of different DIR algorithms. The performance of the DIR algorithms depends strongly on the image artifacts in the different CT‐imaging modalities induced by motion. In CBCT, DIR algorithms produced successfully the volumes and shapes of the stationary targets without producing accurate CT‐numbers. In HCT, the DIR algorithms produced the CT‐number values, lengths, and shapes of the stationary targets even at large motion amplitudes. The ACT images had large image artifacts at large motion amplitudes. The different DIR algorithms failed to produce the shapes, volumes, and CT‐number values of the stationary targets. Thus, the use of deformed CT images from different algorithms and imaging modalities in treatment planning and dose calculation for cancer patients treated with radiation therapy should be evaluated carefully.

## CONFLICT OF INTEREST

There is no conflict of interest.
